# Postoperative eye care in Botswana: the role of ophthalmic nurses

**Published:** 2020-12-31

**Authors:** Chatawana Molao, Tatowela Mmoloki

**Affiliations:** 1 Head of Ophthalmic Nursing Programme, Institute of Health Sciences Molepolole, Botswana.; 2 Ophthalmic Nurse: Tutume Primary Hospital, Botswana.

**Ophthalmic nurses in Botswana play a critical role in the postoperative care of eye patients at tertiary level and in the community.**

**Figure:**
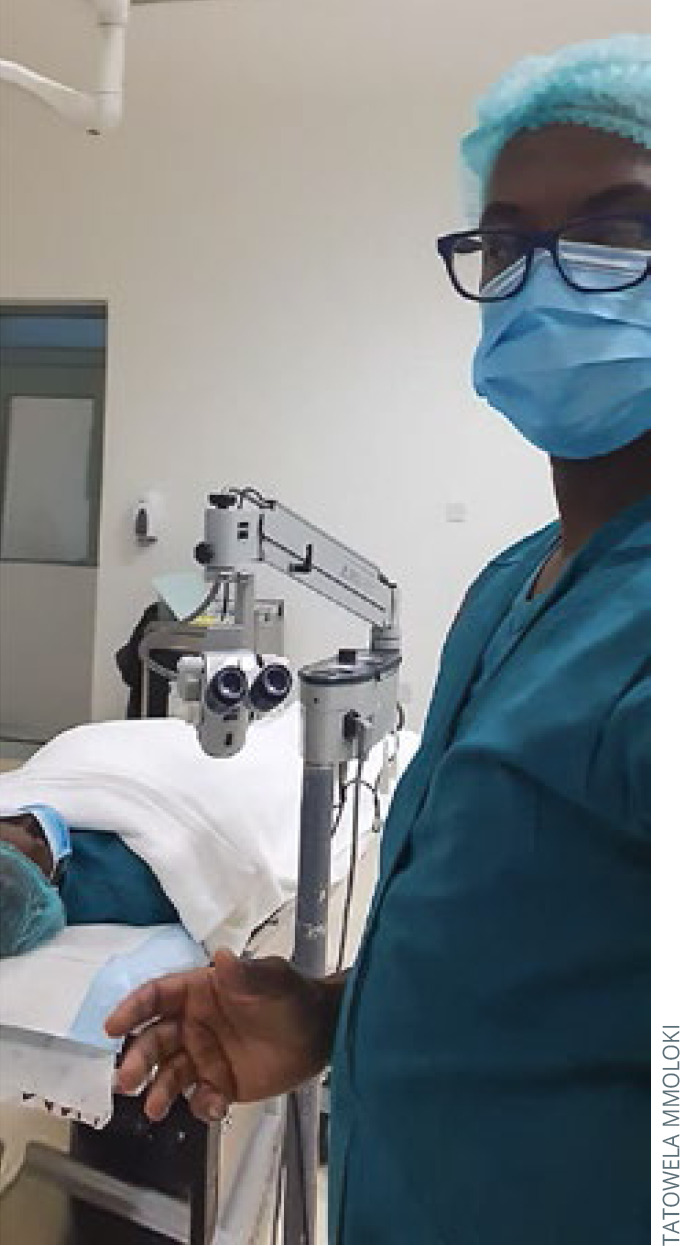
Ophthalmic nurses start discusssing the planned operation and postoperative care with patients on the day of admission. **BOTSWANA**

Ophthalmic nurses make up more than 80% of all the ophthalmic personnel in Botswana. They are usually the first eye care professional a patient will see.

Ophthalmic nurses provide special ophthalmic support to community health clinics within each district (primary care). In district hospitals (secondary level), they independently run eye clinics that provide specialist eye services.

Ophthalmic nurses are also involved in providing tertiary level eye care at Botswana's two centres of excellence for eye health: one in Molepolole village and catchment area and one in Serowe village and catchment area. The two centres are staffed by ophthalmologists, ophthalmic nurses and optometrists. Their services include, but are not limited to, surgical services for cataract, glaucoma, eye injuries, paediatric and optometry services, and other special clinics such as diabetic retinopathy and glaucoma. The ophthalmologists perform most of the major operations carried out at the centres. On discharge, the subsequent postoperative care is carried out by ophthalmic nurses at secondary level and in primary care settings.

## Postoperative management

Ophthalmic nurses play a critical role in managing all eye patients postoperatively. This management follows the nursing process, which entails assessment, planning, implementation, evaluation of the patient's condition at admission until they are discharged, and follow-up care to monitor the progress of the patient's condition.

It is essential to plan postoperative care in advance and discuss it with the patient and significant others before surgery.

## Immediate postoperative ophthalmic care in the ward

After major ophthalmic surgery, ophthalmologists make an initial assessment and decide whether the patient can be discharged or not, depending on the outcome of surgery. Ophthalmic patients who require more observation in the ward are cared for by ophthalmic nurses. The nursing care they provide includes educating patients about their condition, assessing the eyes for signs of complications, and administering medications according to the instructions of the ophthalmologist. This continues until the patient is discharged from the hospital.

## Discharge planning

Discharge planning begins on the day of admission. The ophthalmic nurses plan a meeting with the patients and their family members or companions to discuss the planned operation, possible outcomes, immediate postoperative care while in hospital, and their postoperative care while at home.

## Postoperative patient education

**Figure 1:**
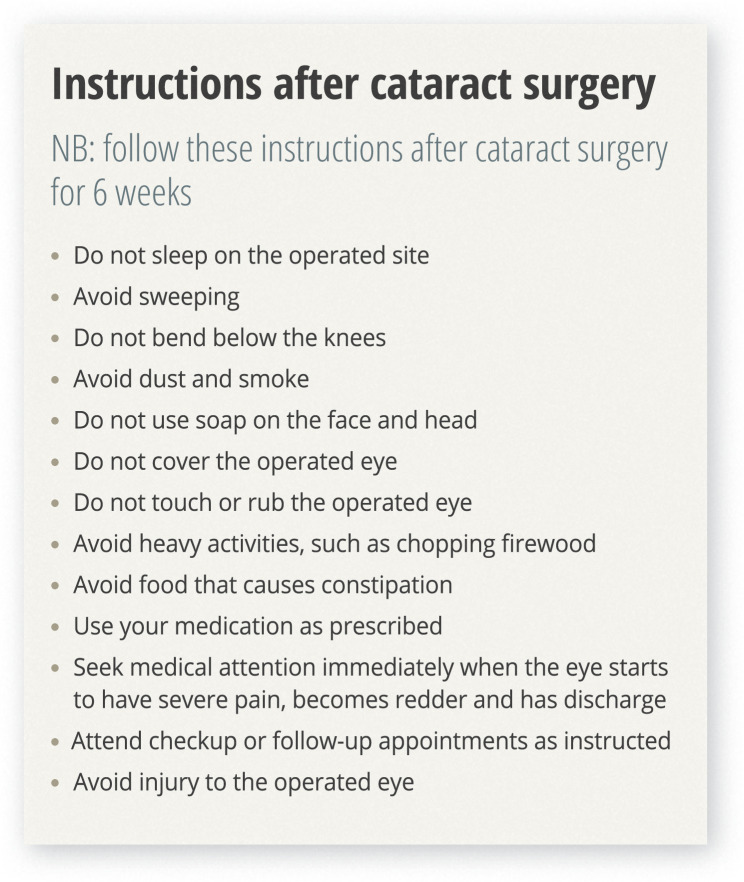
Patients take a copy of these instructions home after surgery.

Special postoperative education depends on the type of surgery. The emphasis is placed on the care of eyes at home, administration and care of eye medications, prevention and recognition of early or late complications, and follow-up schedules. Ophthalmic nurses educate patients on possible key symptoms such as pain, loss of vision and abnormal eye discharge so that they know when to seek help. Patients and their family members/significant others are given health education prior to discharge and are also given an instruction sheet (Figure 1) explaining how to look after their operated eye(s) after surgery. This is attached to the outpatient cards so they can be shared with others at home.

Where appropriate, patients are told to visit the ophthalmic nurses at their local or district hospital for follow-up appointments.

## Postoperative ophthalmic care after discharge

Ophthalmic nurses continue to monitor the patients' condition after surgery at secondary care settings. Patients are usually reviewed at the eye clinic after two weeks to monitor the outcome of surgery; however, patients can be reviewed earlier than two weeks if they experience problems. This ensures prompt referral to the ophthalmologist for further management so that the consequences of the complications are reduced or mitigated.

Eye patients are invited to share their mobile phone numbers and that of their significant others so that they can receive reminders about planned review appointments. This has enhanced trust between the patient and the eye care system and has improved patients' level of compliance with management regimens, including taking prescribed eye medications and coming back for follow-up or review appointments as specified.

**Figure:**
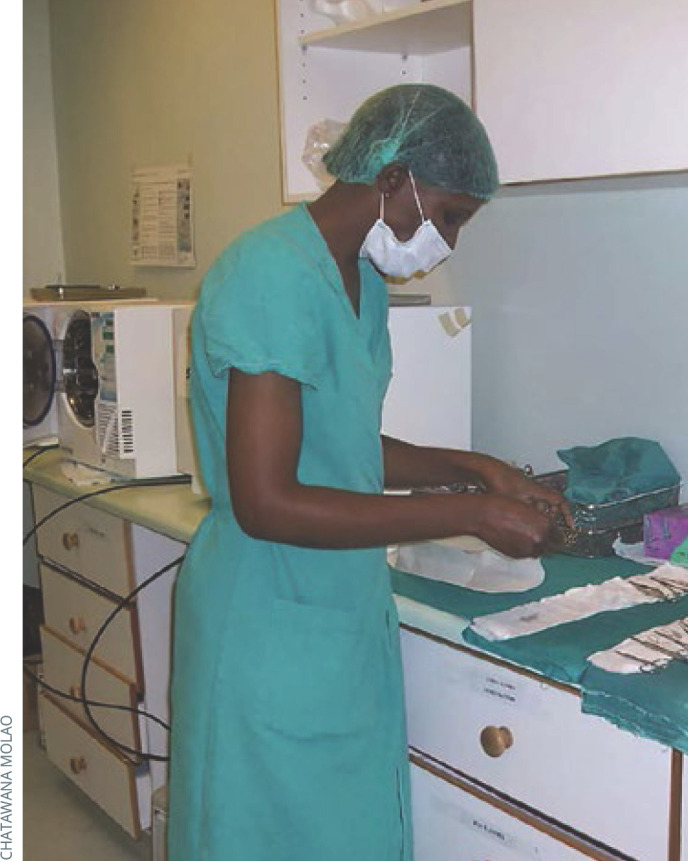
Ophthalmic nurses make an important contribution to the eye health system in Botswana. **BOTSWANA**
